# Influence of intensity ultrasound on rheological properties and bioactive compounds of araticum (*Annona crassiflora*) juice

**DOI:** 10.1016/j.ultsonch.2024.106868

**Published:** 2024-04-03

**Authors:** Jhenifer Cristina Carvalho Santos, Jefferson Luiz Gomes Correa, Maria Luiza Bianchetti Furtado, Larissa Carolina de Morais, Soraia Vilela Borges, Cassiano Rodrigues de Oliveira, Jaime Vilela de Resende, Letícia Fernandes de Oliveira

**Affiliations:** a Department of Food Science, Universidade Federal de Lavras, Lavras, MG, Brasil; b Laboratory of bioprocesses and metabolic biochemistry, Universidade Federal de São João del-Rei, Campus CCO, Divinópolis, MG, Brasil; c Department of Food Science, Universidade Federal de Viçosa, Rio Paranaíba, MG, Brasil

**Keywords:** Response surface methodology, Ultrasound-assisted extraction method, Phenolic compounds, Carotenoids

## Abstract

The use of extracts rich in bioactive compounds is becoming increasingly common in the food, cosmetics, and pharmaceutical industries for the production of functional products. Araticum is a potential fruit to be analyzed due to its content of phenolic compounds, carotenoids and vitamins, with antioxidant properties. Therefore, this study aimed to investigate the effect of ultrasound on total phenolic compounds, total carotenoids, ascorbic acid, color, turbidity and rheology in araticum juice. Response surface methodology based on a central composite design was applied. Araticum juice was subjected to sonication at amplitude levels ranging from 20 to 100 % of the total power (400 W) at a constant frequency of 20 kHz for different durations (2 to 10 min). Morphological analysis was conducted to observe microscopic particles, and viscosity and suitability to rheological models (Newtonian, Power Law, and Herschel-Bulkley) were assessed. The ultrasonic probe extraction method was compared to the control juice. According to the responses, using the desirability function, the optimal conditions for extraction were determined to be low power (low amplitude) applied in a short period of time or low power applied in a prolonged time. These conditions allowed an ultrasonic probe to act on releasing bioactive compounds without degrading them. All three rheological models were suitable, with the Power Law model being the most appropriate, exhibiting non-Newtonian pseudoplastic behavior.

## Highlights

• Higher total phenolic compounds values can be achieved when the processing time was longer (>8 min) and lower power intensity (<80 W) were applied.
• Elevated values of total carotenoids can be obtained when the processing time was longer (>10 min) and power intensity was lower (<150 W) or when the time was shorter (<2 min) and power intensity was higher (>350 W).
• Rheology demonstrates that the extracts were considered pseudoplastic, with assay 4 tending toward Newtonian behavior.
• The conditions that would best provide the desired responses are: low power and short duration, as well as low power and extended duration.

## Graphical abstract

**Figure:**
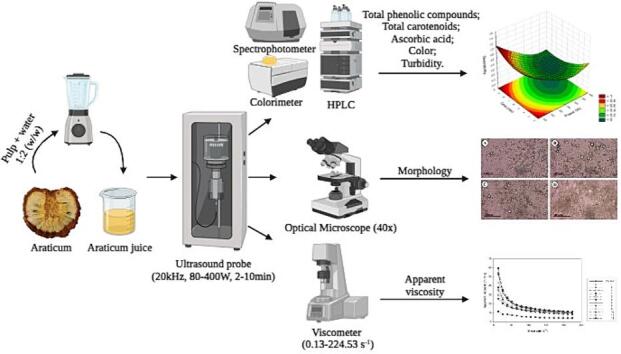

## 1 Introduction

Araticum (*Annona crassiflora*), also popularly known as marolo, ariticum, araticum-do-cerrado, pinha-do-cerrado, is a fruitnative and endemic to the Brazilian Cerrado, belonging to the Annonaceae family. It is a rounded fruit, yellowish-green in color, with thick skin and yellow flesh surrounding the seeds [1], [2]. It is rich in bioactive compounds such as phenolic compounds, carotenoids and ascorbic acid, which promote antioxidant activity, and has a significant nutritional composition, with high levels of moisture, dietary fiber, and carbohydrates. It has soft and sweet pulp, juicy and excellent for the preparation of beverages, ice creams, sweets, preserves and jams [3].

Actually, the demand for nutritious foods with high concentrations of bioactive compounds, quality, quantity, and ease of consumption has significantly increased, prompting modifications in processing techniques [4]. The use of emerging technologies such as ultrasound is a growing field of research that will continue to play a vital role in the food industry [5].

Ultrasound-assisted extraction is a technique that uses ultrasound waves to rupture plant cell walls. It offers several benefits, including higher extraction efficiency, better quality, and higher yield compared to conventional methods [6]. Recently, this technique has been employed to extract bioactive compounds from various fruits and vegetables, potentially improving the bioavailability of various bioactive compounds and enhancing the nutritional content of fruit juices [7], [8]. However, despite the widespread use of conventional extraction techniques, there is still a notable gap in literature regarding the extraction of bioactive compounds and carotenoids from *Annona crassiflora* juice [1], [3].

The use of ultrasound is a promising technique, with its primary effect being cavitation, which involves the formation of bubbles in the liquid that, upon collapse, generate microjets of solution that penetrate into the solid [9]. It also causes changes in the pressure conditions of the food and the liquid immersion medium. Rapid compressions and expansions occur within the food matrix, a phenomenon known as the sponge effect. This effect leads to an increase in mass diffusivity, both for moisture and solutes, through the expulsion of internal gases and the entry of solution into the food pores [10]. However, the process variables such as ultrasound power intensity, frequency, temperature, or pressure, as well as the characteristics of the medium, viscosity, food matrix composition, and the type of interactions and chemical reactions could affect the final quality of the product. [11].

Therefore, the aim of this study was to assess the effects of ultrasound-assisted probe extraction, optimizing process conditions (power and time) that affect the yields of bioactive compounds (carotenoids and ascorbic acid) and rheology of araticum juice using response surface methodology. Aiming to determine the best process and extract for potential application in food, cosmetics, and pharmaceutical products.

## 2 Materials and methods

### 2.1 Material and juice preparation

Araticum (*Annona crassiflora*) fruits were obtained in Belo Horizonte, MG state, Brazil. The fruits were washed, sanitized (200 mg/L sodium hypochlorite for 15 min) and pulped. The pulp was stored in a freezer at −18 °C until the extractions were performed. The conventional extraction was prepared by diluting the pulp in distilled water (1:2 w/w) and blending it in a semi-industrial 60 Hz blender (Siemsen, LS-06, Brazil), followed by filtration through a nylon filter. this juice, obtained through a conventional extraction method, was used as a control.

### 2.2 Sonication extraction

A 400 W digital Sonifier (Model 400, Branson, Danbury, U.S.A.) with a 1.3 cm diameter probe tip was used for sonication of the juice. Samples, obtaining by conventional extraction, were processed at a constant ultrasound frequency of 20 kHz. Araticum juice samples (150 mL) were subjected to ultrasound probe, submerged to a depth of 25 mm in the sample. Extrinsic parameters of power and time were varied according to an experimental design. Power levels were adjusted from 20 % to 100 % of the total input power (400 W). Due to the heat generated by ultrasound, short processing times (2 to 10 min) were applied [12].

### 2.3 Experimental design

A central composite rotatable design (CCRD) was used to design the experiments for araticum juice sonication using two factors: power and time. Five levels of each variable were chosen for the study, including the central point and two axial points. A total of 12 combinations were performed, including four repetitions of the central point (Table 1).

**Table 1:** Real and coded independent variables of the CCRD for ultrasonic probe extraction.

Power (W)	80 (-1.41)	127 (-1)	240 (0)	353 (1)	400 (1.41)
Time (min)	2 (-1.41)	3 (-1)	6 (0)	9 (1)	10 (1.41)

Coded values are between ().

It was assumed that there exists a mathematical function for the response variable Y (phenolic compounds, carotenoids, ascorbic acid, total color difference, soluble solids, and turbidity), in terms of two independent process variables [13], power (X_1_), and processing time (X_2_):

(1) $$Y=\beta_{0}+\beta_{1}X_{1}+\beta_{2}X_{2}+\beta_{11}X_{1}^{2}+\beta_{22}X_{2}^{2}+\beta_{12}X_{1}X_{2}$$

The Statistica 14.1.0.8 software package was used to obtain the regression coefficients, analysis of variance (ANOVA), lack of fit test, and generation of three-dimensional graphs. The quality of the model fit is expressed by the coefficient of determination R^2^, and by ANOVA. The main part of the ANOVA table is used for F-testing the model against the residual, the F-value most be higher than F-tabulated, implying that the model presents good adequancy and fitness to experimental data. In the results, the regressions were presented in a coded form. The optimization was concurrently performed for both response variables using the Desirability function.

### 2.4 Determination of total phenolic compounds

The analysis of total phenolic compounds was conducted following the adapted Folin-Ciocalteu method [14]. Diluted extracts (0.5 mL) were mixed with 2.5 mL of Folin-Ciocalteu reagent (10 %) and 2 mL of sodium carbonate solution (4 %). The mixture was stirred and kept at room temperature for 2 h in the dark. Absorbance was measured at 750 nm. Aqueous solutions of gallic acid were used to construct the standard curve. The results were expressed in mg of gallic acid equivalent (GAE) per gram of the sample. All measurements were performed in triplicate.

### 2.5 Determination of total carotenoids

Total carotenoids were extracted and determined according to the method described by [15]. Ten grams of the sample were homogenized in 40 mL of an extracting solution of isopropyl alcohol: hexane (3:1). The content was transferred to a 125 mL separation funnel wrapped in aluminum foil, to which 50 mL of distilled water was added, and allowed to rest for 30 min followed by three filtrations. Filtration was performed using cotton impregnated with anhydrous sodium sulfate, and the solution was collected in an amber 50 mL volumetric flask, to which 5 mL of acetone was added, and then the volume was made up with hexane. The absorbance was measured at 450 nm, and the results were expressed in mg of total carotenoids per 100 g of the sample, as described in the equation:

(2) $$\mathrm{Total}\;\mathrm{carotenoids}=\frac{A\times 100}{250\times L\times W}$$

where A is the measured absorbance, L is thecuvette width and W, the original quotient between the initial sample and the final dilution volume.

### 2.6 Determination of ascorbic acid

The extraction with ascorbic acid was performed following the methodology described by [16], with slight modifications. Approximately 5 g of the sample was weighed and 10 mL of metaphosphoric acid solution (4.5 %) in ultrapure water were added, allowing it to stand for 1 h in an amber flask. The sample was subsequently filtered using filter paper, and the supernatant was centrifuged at 7,000 rpm for 10 min and transferred to a 1.5 mL flask. This was placed in an ultrasonic bath (Unique brand, model USC 2850 A, Indaiatuba, Brazil) for 30 min. A high-performance liquid chromatograph (HPLC, Shimadzu, LC-20AT) equipped with a UV detector (Shimadzu, SPD-20A) was used to determine the content of ascorbic acid in the samples following [17] with modifications. The separation was performed on a Phenomenex 5 μm C18 column (250 x 4.6 mm), thermostated at 30 °C. The mobile phase was an aqueous solution of 0.15 % acetic acid (v/v) with a flow rate of 1.0 mL min-1. Detection was carried out at 254 nm. The ascorbic acid peak was identified by its retention time compared to standard solutions. The analytical curve was obtained from the chromatogram of the standards by measuring the peak areas of ascorbic acid under the same separation conditions applied to the samples. The standard concentrations of ascorbic acid ranged from 1 to 600 mg/L.

### 2.7 Color

Color determination was performed using a colorimeter (model CM5, Konica Minolta Spectrophotometer), operating in the CIE LCH system to measure the parameters. Reflectance instruments determined the color parameters: L* (lightness or brightness), a* (red/green), and b* (yellow/blue). The numerical values of L*, a*, and b* were converted to TCD (total color difference), which indicates the magnitude of the color change after treatment, using Eq. (3), according to [18]. The reference value for TCD was the non-sonicated juice.

(3) $$TCD=\sqrt{{\left(L^{*}-L_{0}^{*}\right)}^{2}+{\left(a^{*}-a_{0}^{*}\right)}^{2}+{\left(b^{*}-b_{0}^{*}\right)}^{2}}$$

The subscript 0 in Eq. (3) signifies the control sample (untreated).

### 2.8 Emulsions

Samples from the extraction and control experiments had an adjuvant (maltodextrin DE10) added at a percentage of 15 % in relation to the soluble solids of the extract to analyze the turbidity and morphology of the samples. These additional analyses were conducted to assess turbidity and morphology for potential further spray drying [19], [20], [21].

#### 2.8.1 Total soluble solids (°Brix)

The total soluble solids content was measured at 20 ± 0.5 °C using a digital refractometer (HI 96801, Hanna) [22].

#### 2.8.2 Turbidity

Turbidity analysis by spectrophotometry was performed according to [23], using a 1.0 mL aliquot of the emulsions dispersed in 100 mL of distilled water, with a 3 mL sample used for spectrophotometric reading, conducted in a quartz cuvette with an optical path length of 1.00 cm. The wavelength analyzed was 600 nm.

(4) $$T=\frac{2,303\times A_{\lambda }\times fd}{l}$$

where A_λ_ is the absorbance value of the emulsion at 600 nm, fd is the dilution factor applied to the emulsion and l (cm) is the optical path length of the cuvette.

#### 2.8.3 Optical microscopy

Stability analyses based on micrographs were performed by visually assessing the dispersions, where characteristics such as particle size increase, flocculation, and coalescence were observed for the different assays. To conduct optical microscopy of the emulsions, a drop of the extract was placed on a glass slide, and a cover slip was then positioned over the drop. The sample was observed using a BA210E optical microscope (Motic) equipped with an attached tablet. The optical magnification was set at 40x. Image processing was performed using CorelDRAW 2018 (64 Bit) software.

### 2.9 Rheology

The rheological profile of araticum extracts was determined at 25 °C using a concentric cylinder rotational viscometer (Brookfield DVIII Ultra, Brookfield Engineering Laboratories, USA). The analyses were carried out with three repetitions employing the SC4-18 spindle and a gradually increasing shear rate ranging from 0.13 to 224.53 s^−1^. To determine the flow profile of the fluids, the Power Law model (Equation 1) was fitted to the shear stress and shear rate data [24].

(5) $$\sigma =k{\dot{\gamma }}^{n}$$

Where σ is the shear stress (Pa), k is the consistency index (Pa.s^n^), $\dot{\gamma }$ is the shear rate (s^−1^), and n is the flow behavior index (dimensionless).

## 3 Results and discussion

Table 2 presents the results of the analyzed parameters in the CCRD experimental design for Araticum juice.

**Table 2:** Results of total phenolic compounds (TPC), total carotenoids (TC), ascorbic acid (AA), color parameters (b*, TCD) and turbidity, from the araticum juice extraction experiments according to the CCRD experimental design.

Treatments	X_1_^**^	X_2_^**^	TPC (mg GAE/g)	TC (mg/ 100 g)	AA (mg/L)	Color parameters		Turbidity (T/cm^−1^)
						b*	TCD	
Control	–	–	9.59	2.85	4.78	36.07	–	129.83
1	127	3	8.98	2.85	3.57	34.54	1.98	132.60
2	353	3	6.10	3.18	3.81	33.51	2.93	131.44
3	127	9	10.82	2.98	3.90	34.30	2.34	121.66
4	353	9	4.87	2.80	3.75	32.84	3.53	132.83
5	80	6	11.75	3.03	4.52	35.52	1.18	133.86
6	400	6	4.84	2.68	4.06	33.08	3.30	129.77
7	240	2	5.44	4.15	4.50	34.92	1.72	125.28
8	240	10	5.58	3.86	4.14	33.82	2.41	132.42
9	240	6	4.84	1.96	4.47	33.62	2.85	119.35
10	240	6	4.81	2.01	4.59	33.80	2.90	119.18
11	240	6	4.95	2.10	4.64	33.93	2.90	118.09
12	240	6	4.94	2.21	4.62	33.80	2.87	118.37

^**^Variables: X_1_: ultrasound power (W); X_2_: time of ultrasound application (min).

### 3.1 Extraction method

The extraction method can directly affect the quality and composition of the obtained extract. On one hand, energy-intensive, and variable-controlled technologies and processes can result on higher extraction yelds [25]. On the other hand, simpler extraction methods could offer lower cost and ease operation. The results indicate that the controlled, simple extraction method yields values close to or even higher than the obtained in some assays. Demonstrating that conventional extraction is efficient depending on the objective, however, extraction using an ultrasonic probe at adequate power and time presents better results for total phenolic compounds and total carotenoids.

### 3.2 Total phenolic compounds (TPC)

The TPC are an important class of phytochemicals formed from secondary substances. Quantifying them in the extract provides information about their bioactivity and the product's quality for nutrition after consumption [1]. The quantification of TPC in the fresh araticum juice and the responses of each execution in the experimental design are presented in Table 2, and the coded equation obtained is provided below. The analysis of variance for the model showed significance (p < 0.05) for the variable power in a negative linear and positive quadratic manner, for the variable time in a positive quadratic manner, and for the interaction between the variables in a negative manner. It exhibited a coefficient of determination of 98 %; therefore, the model provides an approximation to the true system in the equation:

(5) $$\mathrm{TPC}=4.86-2.32X_{1}+1.89X_{1}^{2}+0.11X_{2}+0.59X_{2}^{2}-0.77X_{1}X_{2}$$

**Fig. 1:**
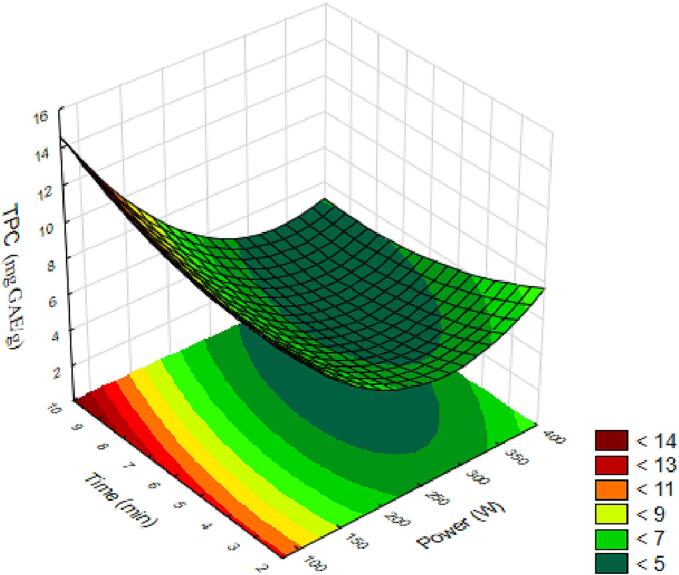
Response surfaces for the total phenolic compounds (TPC) content in araticum juice as a function of power and processing time.

The generated surface is shown in Fig. 1. Observing the response surface, we can notice that the highest amount of total phenolic compounds is obtained with low power and longer ultrasound time. Therefore, higher TPC values can be achieved when the processing time was longer (>8 min) and lower power intensity (<80 W) were applied.

Ultrasonic waves result from the conversion of electrical energy into mechanical energy through piezoelectric materials. When ultrasonic energy propagates in the liquid, cavitation bubbles are formed due to pressure changes. These bubbles violently collapse in subsequent cycles of compression as the sound wave travels, resulting in regions of high temperature and pressure [11]. High power degrades phenolic compounds that are sensitive to temperature and pressure. However, at low power and extended time, the formation of microfractures and microcavitation occurs in plant tissues due to the strong shear forces caused by bubble collapse, leading to cell wall rupture and the release of bioactive compounds [26], [27], [28]. This effect is also observed in the total carotenoids results.

### 3.3 Total carotenoids (TC)

The TC are considered a highly diverse group of natural liposoluble pigments responsible for the red, orange, and yellow colors synthesized by plants, microorganisms, and vertebrates [29]. Although there are few reports on the content and composition of carotenoids in araticum fruits, the literature is consistent in mentioning that the consumption of 100 g of araticum pulp is sufficient to meet the recommended daily intake of vitamin A [30]. Quantifying them in the extract provides concrete information on its potential use in daily consumption. The analysis of variance for the model showed significance (p < 0.05) for the time variable in a positive quadratic manner, as with total phenolic compounds, and exhibited a coefficient of determination of 86 %. Therefore, the model provides the equation:

(6) $$\mathrm{TC}=2.09-0.04X_{1}+0.24X_{1}^{2}-0.08X_{2}+0.90X_{2}^{2}-0.13X_{1}X_{2}$$

**Fig. 2:**
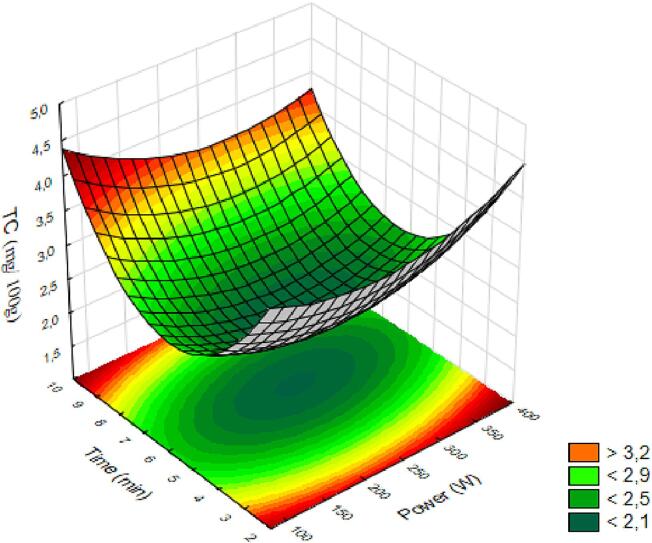
Response surfaces for total carotenoids (TC) in araticum juice as a function of power and processing time.

The generated surface is presented in Fig. 2. When observing the generated surface, lower power and longer time, as well as higher power and shorter time, indicate a higher quantity of total carotenoids. Elevated values of total carotenoids can be obtained when the processing time was longer (>10 min) and power intensity was lower (<150 W) or when the time was shorter (<2 min) and power intensity was higher (>350 W).

We can observe that, as was the case with total phenolic compounds, in the case of carotenoids, low power associated with extended time favors carotenoids. However, in this case, high power for a short period of time also facilitates their extraction, indicating two favorable process options for obtaining the compound. Many ultrasonic processes in fruit products show a trend of initially increasing the concentration of bioactive compounds, followed by a decrease after extended sonication periods. This trend is often related to the initial release of membrane-bound or apoenzyme-bound compounds, followed by the degradation of these compounds by reactive oxygen species produced during sonication [8].

### 3.4 Ascorbic acid (AA)

The AA, also known as vitamin C, is a water-soluble vitamin and an essential nutrient for the human body. It plays several important roles in the organism: antioxidant activity, assists the immune system, collagen synthesis, and non-heme iron absorption. The analysis of variance for the model did not show significance (p < 0.05) for any of the variables and presented a coefficient of determination of 52 %. Therefore, the model was not applied. Upon analyzing the results and noticing that there was no significant difference, we can conclude that the extraction process using ultrasonic probe, regardless of the applied power and time, did not affect the amount of ascorbic acid in the extract.

**Fig. 3:**
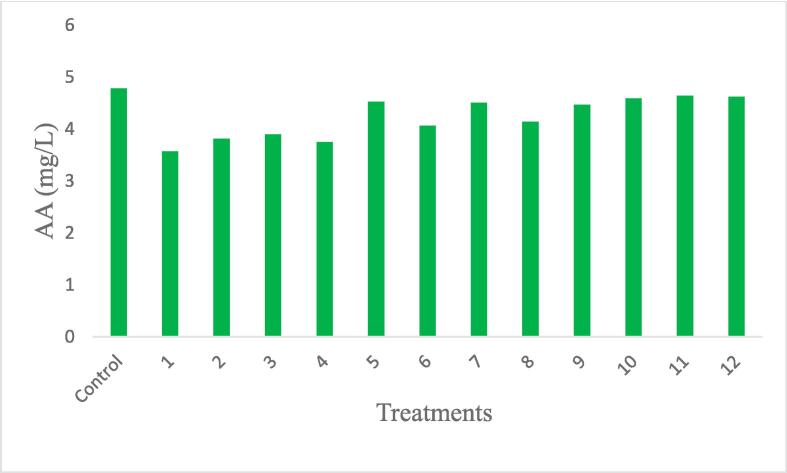
Medium ascorbic acid (AA) for each tested condition.

We can observe that, similar to total carotenoids, power does not significantly interfere with ascorbic acid. However, upon observing Table 2, we notice that higher powers tend to result in a slight degradation of vitamin C, which can also be seen in Fig. 3. This suggests that extraction using an ultrasonic probe facilitates the process, yet high powers and extended periods of sonication may degrade compounds due to reactive oxygen species produced during sonication [8].

### 3.5 Total color difference (TCD) and b*

Color is a visual indicator used to assess the quality of fruit juices and plays a significant role in consumer sensory avaliation [31]. It can reflect the level of acceptance and serve as an indicator of microbial quality during processing and storage [32]. TCD indicates the difference that occurred during the use of the ultrasound probe in comparison to the control. The b* coordinate represents the variation between yellow and blue. The analysis of variance for the models showed significance (p < 0.05) for the power variable in a positive linear manner for total color difference and in the power term also in the linear manner, but negatively for b*, with coefficient of determination of 85 % and 86 %, respectively. Therefore, the models provide the coded equations:

(7) $$\mathrm{TCD}=2.86+0.64X_{1}-0.17X_{1}^{2}+0.25X_{2}-0.27X_{2}^{2}+0.06X_{1}X_{2}$$

(8) $$b^{*}=33.80-0.74X_{1}+0.11X_{1}^{2}-0.31X_{2}+0.15X_{2}^{2}-0.11X_{1}X_{2}$$

**Fig. 4:**
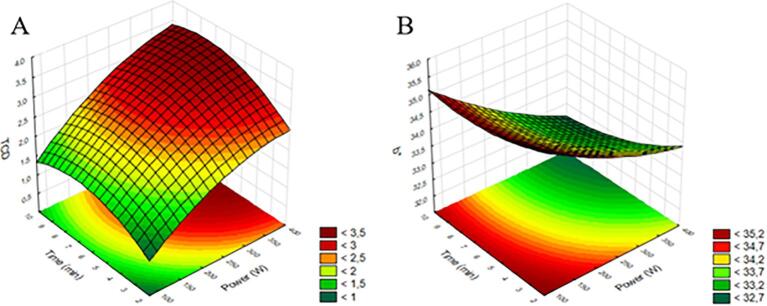
Response surfaces for TCD of araticum juice as a function of power and processing time (A) and response surfaces for the b* parameter of araticum juice as a function of power and processing time (B).

The generated surfaces are shown in Fig. 4. Acording the surface it is possible infer that the smallest difference is observed whith powerbelow 200 W, regardless of the application time. In the same way, it is possible to see that b* is more similar to control, higher b* can be found with power below 200 W, regardless of the processing time, indicating the preservation of the yellow color.

According to [33], it was suggested that sonication is a useful approach for preserving bioactive compounds and preventing the degradation of the light red dragon fruit juice's color. Ultrasound can inhibit enzymes such as polyphenoloxidase and peroxidase, which are responsible for the fruit browning reaction [34]. According to Abid et al. [22], ultrasound treatment results in significant changes in the color of the treated samples. Changes in color during ultrasonic cavitation cause physical, chemical, and biological alterations, such as increased diffusion rates and the decomposition of particles like enzymes and microorganisms [36]. Therefore, its controlled use assists in color preservation.

### 3.6 Turbidity (T)

Turbidity is how the presence of particles affects the propagation of light in the solution. The analysis of variance for the model showed significance (p < 0.05) for the power and time variables in a positive quadratic manner, with a coefficient of determination of 82 %. Therefore, the model provides an approximation to the true system through the equation:

(9) $$T=118.79+0.53X_{1}+6.19X_{1}^{2}-0.004X_{2}+5.27X_{2}^{2}+3.08X_{1}X_{2}$$

**Fig. 5:**
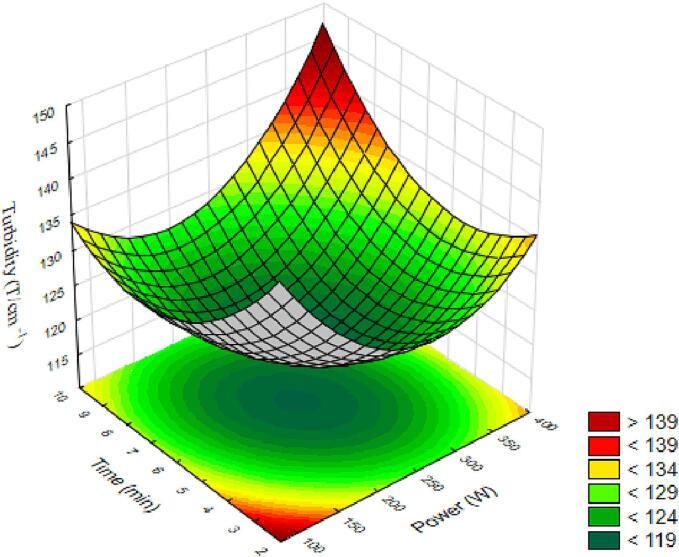
Response surfaces for turbidity of araticum juice as a function of power and processing time.

The generated surface is presented in Fig. 5. When observing the surface, we can note that lower power and time, as well as higher power and longer time, result in higher turbidity values. Elevated values can be achieved when the processing time is longer (>9 min) and power intensity is higher (>400 W), or when the time is shorter (<2 min) and power intensity is lower (<100 W). The increase in the cloud point of ultrasound treatments compared to control samples is likely due to the high-pressure gradient caused by cavitation during ultrasound treatment [35]. According to the findings of [22], ultrasound treatment significantly increased the cloud point in apple juice (p < 0.05). Walkling-Ribeiro et al. [36] observed that the effect of ultrasound and pulsed electric treatments was significant on the cloud point of grapefruit.

### 3.7 Morphology

**Fig. 6:**
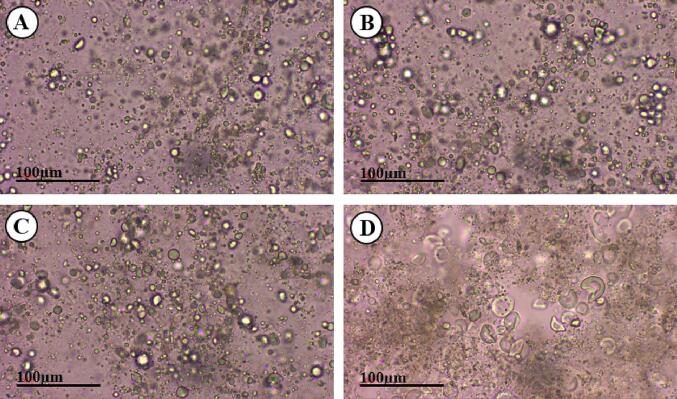
Optical micrograph of araticum juice. A - Control without maltodextrin; B - Control with maltodextrin; C - Assay 5; and D - Assay 4.

The morphology of solutions is a visual analysis of the microscopic characteristics observed after a process, examining how it affects the cells and particles dispersed within the medium. In Fig. 6, we can observe that image A shows araticum juice, a control without maltodextrin, with dispersed particles in the medium. In image B, the control with the addition of maltodextrin, one can see that the particles are slightly more agglomerated, which is also noticeable in image C (assay 5), where the lowest power was used. This indicates that low powers did not provoke visible changes in the morphology of araticum juice and the maltodextrin added to encapsulate the compounds brought them closer together. However, in image D, the particles are shattered due to the high power of the ultrasound probe for an extended period in the emulsion (assay 4), causing the rupture of plant cells due to cavitation. Studies have asserted that collapsing bubbles become stronger at higher amplitudes because the bubble size is directly proportional to the amplitude of the ultrasound waves [26]. According to research conducted by [37] on a microscopic scale, ultrasound increases turbidity levels and stabilizes fruit juice by crushing large particles in the juice and creating a stable suspension, thereby enhancing the physical appearance of apple juice. As observed in the turbidity analysis, at high powers for an extended period, there is an accumulation of dispersed particles that hinder the passage of light through the solution, making it more turbid.

### 3.8 Rheology

The fruit juice represents a complex system, composed of two distinct phases: the liquid phase, known as the serum phase, characterized mainly by the presence of water, sugars, soluble vitamins, and other compounds soluble in water; and the dispersed phase, also called pulp, composed of larger and colloidal particles, including soluble/insoluble pectins and fibers [38]. Studies indicate that the rheological properties of fruit juices are not solely attributable to the liquid phase. Variations in the dispersed phase are also associated with these rheological properties [39]. The stability of a suspension is crucially important for process control and is closely related to rheological properties. Throughout various stages of fruit juice production, such as pumping, the rheological properties of the juice are affected [40].

**Fig. 7:**
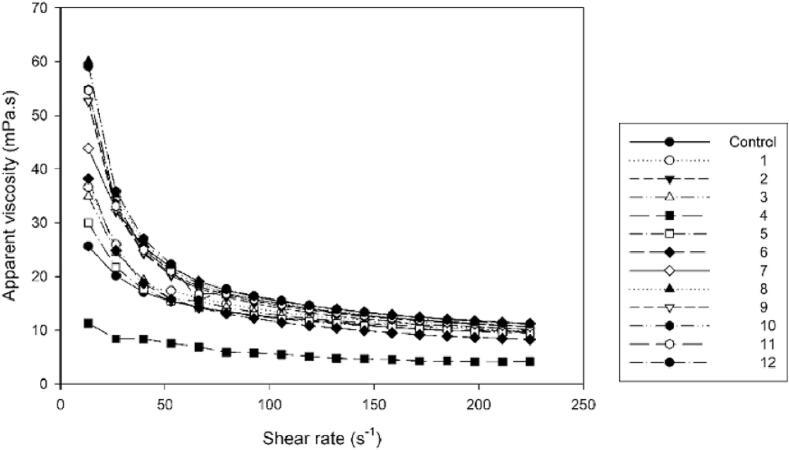
Rheograms of araticum extracts. Relationship between apparent viscosity and shear rate.

The viscoelastic behavior of the juice is caused by a complex interaction among its particles. Several mathematical models (Newtonian, Power-Law, Herschel-Bulkley, and Bingham) describe liquid flow behavior using shear stress and shear rate as parameters. In the rheological analysis of araticum fruit juices, the Newtonian, Power Law, and Herschel-Bulkley models were fitted to the data, with the exception of treatments 2, 8, 10, 11, and 12, which did not fit the Herschel-Bulkley model. The model that best describes the behavior of the samples is the Power Law model, as it exhibited the highest R^2^ and the lowest RMSE error. All treatments displayed pseudoplastic non-Newtonian fluid behavior, as viscosity decreased with increasing shear rate. The pseudoplastic profile is further confirmed by the value of n (flow behavior index), as n is less than 1 for all treatments. The consistency index (k), indicating the fluid's flow resistance, showed that the extracts with lower viscosity are the control treatments, 4, and 5 (Table 3 and Fig. 7). Among these, treatment 4 stands out as the least viscous.

**Table 3:** Parameters of the Power Law model fitted for marolo extracts.

Treatments	k(Pa·s^n^)	*n*	R^2^	RMSE
Control	0.058 ± 0.002	0.669 ± 0.004	0.999	0.010
1	0.098 ± 0.016	0.581 ± 0.028	0.996	0.035
2	0.159 ± 0.026	0.488 ± 0.025	0.987	0.055
3	0.081 ± 0.010	0.608 ± 0.019	0.997	0.031
4	0.035 ± 0.005	0.600 ± 0.030	0.991	0.022
5	0.065 ± 0.006	0.642 ± 0.014	0.998	0.022
6	0.111 ± 0.015	0.517 ± 0.020	0.994	0.033
7	0.145 ± 0.011	0.524 ± 0.010	0.992	0.051
8	0.185 ± 0.021	0.469 ± 0.018	0.982	0.068
9	0.155 ± 0.011	0.509 ± 0.012	0.988	0.058
10	0.164 ± 0.022	0.492 ± 0.021	0.988	0.057
11	0.157 ± 0.003	0.498 ± 0.003	0.988	0.058
12	0.182 ± 0.015	0.478 ± 0.013	0.985	0.064

k is the consistency index (Pa s^n^) and n is the flow behavior index (dimensionless), R^2^ is the coefficient of determination, and RMSE is the root mean square error.

Means followed by the same lowercase letters in the column do not differ significantly from each other according to the Tukey test (p < 0,05).

The consistency index (k) provides information about the material's ability to resist applied shear forces, that is, it indicates the viscosity of the material. The higher the value of k, the greater the material's resistance to deformation, that is, it has greater viscosity. For further processing of extracts, lower viscosities are of interest. Therefore, the smallest values of k are observed in Table 3 at the extremes, that is, higher powers and longer time and lower power and shorter time.

The n value for all treatments was less than 1.0 (Table 3), corresponding to the flow curve, which indicated pseudoplastic properties (0 < n < 1). The values of n decrease using greater power and time, in the same way as with lower power, regardless of time. The closer the n values are to 1, the closer the fluid approaches Newtonian behavior, therefore, values closer to 1 are required.

The flow curves of araticum pulp for the different treatments are shown in Fig. 7. The apparent viscosity in all samples significantly decreased with an increase in shear rate, displaying characteristics of a non-Newtonian fluid.

The low viscosity presented by assay 4 is due to its exposure to high power for an extended period, causing the breakage of particles present in the juice, which can be visibly observed in Fig. 6 (D). The second least viscous treatment is the control, followed by treatment 5, where in this test, lower power was applied for an intermediate period (6 min). A similarity between the treatments can also be observed in Fig. 6. However, treatment 2, which had high power but a short duration, exhibited high viscosity, highlighting that the exposure time to the ultrasonic probe affects the viscosity of araticum juice. In the study of the use of ultrasound in the treatment of açaí juice [41], it shows that ultrasonic processes reduced the viscosity of the juices. The authors associate the high energy of the ultrasound process with the fact that the particles decompose in the food, reducing the viscosity of the juice. This behavior was also observed in the present study.

### 3.9 Optimization

**Fig. 8:**
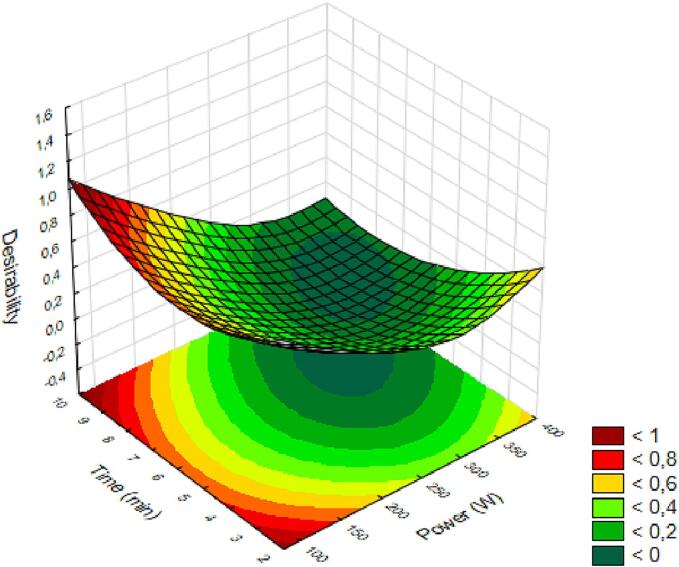
Optimal response surfaces of araticum juice as a function of power and processing time based on the obtained responses.

The optimal surface obtained from the independent variables and desirability, where the ideal is close to 1, indicates that low power and short duration, as well as low power and long duration, provide the best conditions for extracting araticum juice with the desired responses, as shown in Fig. 8. The extraction process using an ultrasonic probe at low power causes the breakdown of plant tissues due to strong shear forces caused by bubble collapse, yet without degrading the bioactive compounds. This results in the release of compounds while avoiding an increase in temperature and pressure caused by high powers in subsequent cycles [11], [22].

## 4 Conclusion

Response Surface Methodology has proven to be an effective technique for investigating the effects of ultrasonic probe power and processing time on total phenolic compounds, total carotenoids, ascorbic acid, total color difference, parameter b*, and turbidity. The coefficient of determination (R^2^) for the predicted models showed good correlation with experimental data, reaching a reliability level of up to 98 % for total phenolic compounds. Rheology demonstrates that the extracts were considered pseudoplastic, with assay 4 tending toward Newtonian behavior. This study demonstrates that the ultrasonic probe significantly influences the bioactive compounds, color, and rheology of araticum juice. The color changes observed during sonication were subtle but significant, indicating alterations in the appearance of the fruit juice. The conditions that would best provide the desired responses are: low power and short duration, as well as low power and extended duration. Although sonication caused degradation of bioactive compounds under certain processing conditions, this technology can be suitable for processing araticum juice with high levels of bioactive compound retention under appropriate conditions.

## CRediT authorship contribution statement

**Jhenifer Cristina Carvalho Santos:** Writing – review & editing, Writing – original draft. **Jefferson Luiz Gomes Correa:** Writing – review & editing, Supervision. **Maria Luiza Bianchetti Furtado:** Methodology. **Larissa Carolina de Morais:** Methodology, Data curation. **Soraia Vilela Borges:** Resources. **Cassiano Rodrigues de Oliveira:** Methodology, Data curation. **Jaime Vilela de Resende:** Resources. **Letícia Fernandes de Oliveira:** Writing – review & editing, Supervision.

## Declaration of competing interest

The authors declare that they have no known competing financial interests or personal relationships that could have appeared to influence the work reported in this paper.

## Data Availability

Data will be made available on request.
